# Contribution of Previously Unrecognized RNA Splice-Altering Variants to Congenital Heart Disease

**DOI:** 10.1161/CIRCGEN.122.003924

**Published:** 2023-05-11

**Authors:** Min Young Jang, Parth N. Patel, Alexandre C. Pereira, Jon A.L. Willcox, Alireza Haghighi, Angela C. Tai, Kaoru Ito, Sarah U. Morton, Joshua M. Gorham, David M. McKean, Steven R. DePalma, Daniel Bernstein, Martina Brueckner, Wendy K. Chung, Alessandro Giardini, Elizabeth Goldmuntz, Jonathan R. Kaltman, Richard Kim, Jane W. Newburger, Yufeng Shen, Deepak Srivastava, Martin Tristani-Firouzi, Bruce D. Gelb, George A. Porter, Christine E. Seidman, Jonathan G. Seidman

**Affiliations:** 1 Departments of Genetics (M.Y.J., P.N.P., A.C.P., J.A.L.W., A.H., A.C.T., S.U.M., J.M.G., D.M.M., S.R.D., C.E.S., J.G.S.), Harvard Medical School, Boston, MA.; 2 Pediatrics (S.U.M.), Harvard Medical School, Boston, MA.; 3 Department of Medicine (M.Y.J., A.H.), Brigham and Women’s Hospital, Boston, MA.; 4 Division of Cardiology (S.R.D., C.E.S.), Brigham and Women’s Hospital, Boston, MA.; 5 Division of Cardiology, Massachusetts General Hospital, Boston, MA (P.N.P.).; 6 Laboratory for Cardiovascular Diseases, RIKEN Center for Integrative Medical Sciences, Yokohama, Japan (K.I.).; 7 Division of Newborn Medicine (S.U.M.), Boston Children’s Hospital, MA.; 8 Department of Cardiology (J.W.N.), Boston Children’s Hospital, MA.; 9 Department of Cardiology (J.W.N.), Boston Children’s Hospital, MA.; 10 Department of Pediatrics, Stanford University, Palo Alto, CA (D.B.).; 11 Departments of Genetics (M.B.), Yale University School of Medicine, New Haven, CT.; 12 Pediatric Cardiology (M.B.), Yale University School of Medicine, New Haven, CT.; 13 Departments of Pediatrics (W.K.C.), Columbia University Medical Center, New York, NY.; 14 Medicine (W.K.C.), Columbia University Medical Center, New York, NY.; 15 Systems Biology (Y.S.), Columbia University Medical Center, New York, NY.; 16 Biomedical Informatics (Y.S.), Columbia University Medical Center, New York, NY.; 17 Cardiorespiratory Unit, Great Ormond Street Hospital, London, United Kingdom (A.G.).; 18 Department of Pediatrics, Perelman School of Medicine at the University of Pennsylvania, Philadelphia, PA (E.G.).; 19 Heart Development and Structural Diseases Branch, Division of Cardiovascular Sciences, National Institute of Heart, Lung, and Blood, National Institutes of Health, Bethesda, MD (J.R.K.).; 20 Department of Cardiac Surgery, Smidt Heart Institute, Cedars-Sinai Medical Center, Los Angeles, CA (R.K.).; 21 Gladstone Institute of Cardiovascular Disease, San Francisco, CA (D.S.).; 22 Nora Eccles Harrison Cardiovascular Research and Training Institute, University of Utah, Salt Lake City, UT (M.T.-F.).; 23 Mindich Child Health and Development Institute (B.D.G.), Icahn School of Medicine at Mount Sinai, New York.; 24 Department of Pediatrics (B.D.G.), Icahn School of Medicine at Mount Sinai, New York.; 25 Department of Genetics (B.D.G.), Icahn School of Medicine at Mount Sinai, New York.; 26 Department of Genomic Sciences (B.D. co-occurrence G.), Icahn School of Medicine at Mount Sinai, New York.; 27 Department of Pediatrics, University of Rochester Medical Center, NY (G.A.P.).; 28 Howard Hughes Medical Institute, Chevy Chase, MD (C.E.S.).

**Keywords:** algorithms, alleles, child, humans, RNA splicing

## Abstract

**Background:**

Known genetic causes of congenital heart disease (CHD) explain <40% of CHD cases, and interpreting the clinical significance of variants with uncertain functional impact remains challenging. We aim to improve diagnostic classification of variants in patients with CHD by assessing the impact of noncanonical splice region variants on RNA splicing.

**Methods:**

We tested de novo variants from trio studies of 2649 CHD probands and their parents, as well as rare (allele frequency, <2×10^−^^6^) variants from 4472 CHD probands in the Pediatric Cardiac Genetics Consortium through a combined computational and in vitro approach.

**Results:**

We identified 53 de novo and 74 rare variants in CHD cases that alter splicing and thus are loss of function. Of these, 77 variants are in known dominant, recessive, and candidate CHD genes, including *KMT2D* and *RBFOX2*. In 1 case, we confirmed the variant’s predicted impact on RNA splicing in RNA transcripts from the proband’s cardiac tissue. Two probands were found to have 2 loss-of-function variants for recessive CHD genes *HECTD1* and *DYNC2H1*. In addition, SpliceAI—a predictive algorithm for altered RNA splicing—has a positive predictive value of ≈93% in our cohort.

**Conclusions:**

Through assessment of RNA splicing, we identified a new loss-of-function variant within a CHD gene in 78 probands, of whom 69 (1.5%; n=4472) did not have a previously established genetic explanation for CHD. Identification of splice-altering variants improves diagnostic classification and genetic diagnoses for CHD.

**Registration:**

URL: https://clinicaltrials.gov; Unique identifier: NCT01196182.

**See Editorial by Blue and Mital**

Congenital heart disease (CHD) is the most common major congenital anomaly, affecting 1 in 100 live births in the United States.^1,2^ CHD causes vary widely, including genetic pathogeneses ranging from monogenic conditions to chromosomal aneuploidies, as well as environmental factors and maternal exposures.^3–5^ While the advent of whole exome sequencing (WES) has allowed identification of pathogenic variants in CHD, up to 60% of CHD cases remain unexplained by known genetic or environmental causes.^4^ Because the genetic and molecular underpinnings of CHD pathogenesis are incompletely understood, we do not know whether these unsolved CHD cases are, in part, due to unrecognized coding and noncoding variants in known and unidentified CHD genes.

Previous analyses of WES from CHD probands, unaffected children, and their parents have shown that there is ≈1 de novo variant (DNV) in the exome of every individual regardless of disease status. However, de novo loss-of-function (LoF) variants were enriched in CHD probands compared with unaffected children in a set of genes, which includes some chromatin modifiers and transcription factors.^6,7^ LoF variants are defined as variants that alter the resultant protein by causing frameshift, nonsense, or alter canonical RNA splice sites (GT in the donor sequence and AG in the acceptor sequence). Other de novo and rare sequence variants in coding and noncoding regions may also have a functional effect, but interpretation of these remains challenging. Many are classified as variants of uncertain significance.

To further define genetic causes in up to 60% of patients with CHD for whom the pathogenesis of CHD is unexplained, we focused on genetic variants that may alter RNA splicing. While alterations to canonical splice sites alter the resultant protein through disruptions in splicing, a subset of variants that occur in noncanonical RNA splice regions can also affect mRNA splicing. Recent computational methods have improved predictions of variants of uncertain significance in splice regions,^8,9^ but the definitive classification of these variants is achieved by analysis of resultant spliced transcripts. In prior studies, we developed an assay that combines in silico and in vitro methods to select variants for their predicted ability to alter splicing, which are then validated using an in vitro human embryonic kidney cellular assay to detect splicing defects in the resultant transcript.^10,11^ This assay, when applied to target genes identified in a cohort of cardiomyopathy patients, increased the yield of identifying damaging splice variants by ≈50%.^10–12^

We hypothesize that some CHD cases could also be explained by genetic variation that alters RNA splicing. Applying our established approach, we assessed both rare variants and DNVs of uncertain significance within a CHD cohort to expand the diagnostic yield of traditional sequencing methods. Uncovering novel LoF variants within noncanonical splice regions aids in establishing a genetic diagnosis for patients with CHD and may facilitate discovery of new genes that are critical for normal cardiovascular development and potentially cause CHD.

## Methods

Full details of the methods used in this study are available online within the Supplemental Material. All data and supporting materials are available within the article and its Supplemental Material. CHD probands were recruited from 2 centers into the Congenital Heart Disease Genetic Network Study of the Pediatric Cardiac Genomics Consortium (CHD genes: NCT01196182), and written informed consent was obtained from each participant or parent/guardian. The protocol was approved by the Institutional Review Boards of Boston Children’s Hospital, Brigham and Women’s Hospital, Great Ormond Street Hospital, Children’s Hospital of Los Angeles, and Yale School of Medicine.

## Results

### Novel RNA Splice-Altering DNVs in CHD Probands and Controls

Among 3156 DNVs identified previously through WES analyses of 2649 CHD probands and their parents, we excluded variants predicted to cause LoF, including nonsense, frameshift, canonical splice sites, and insertions/deletions, as well as variants within single-exon transcripts. Parallel filters were applied to 1913 DNVs identified in WES data in 1789 Simons Foundation Autism Research Initiative controls. This yielded 2687 and 1703 DNVs in the CHD and control cohorts, respectively.

**Figure 1.**
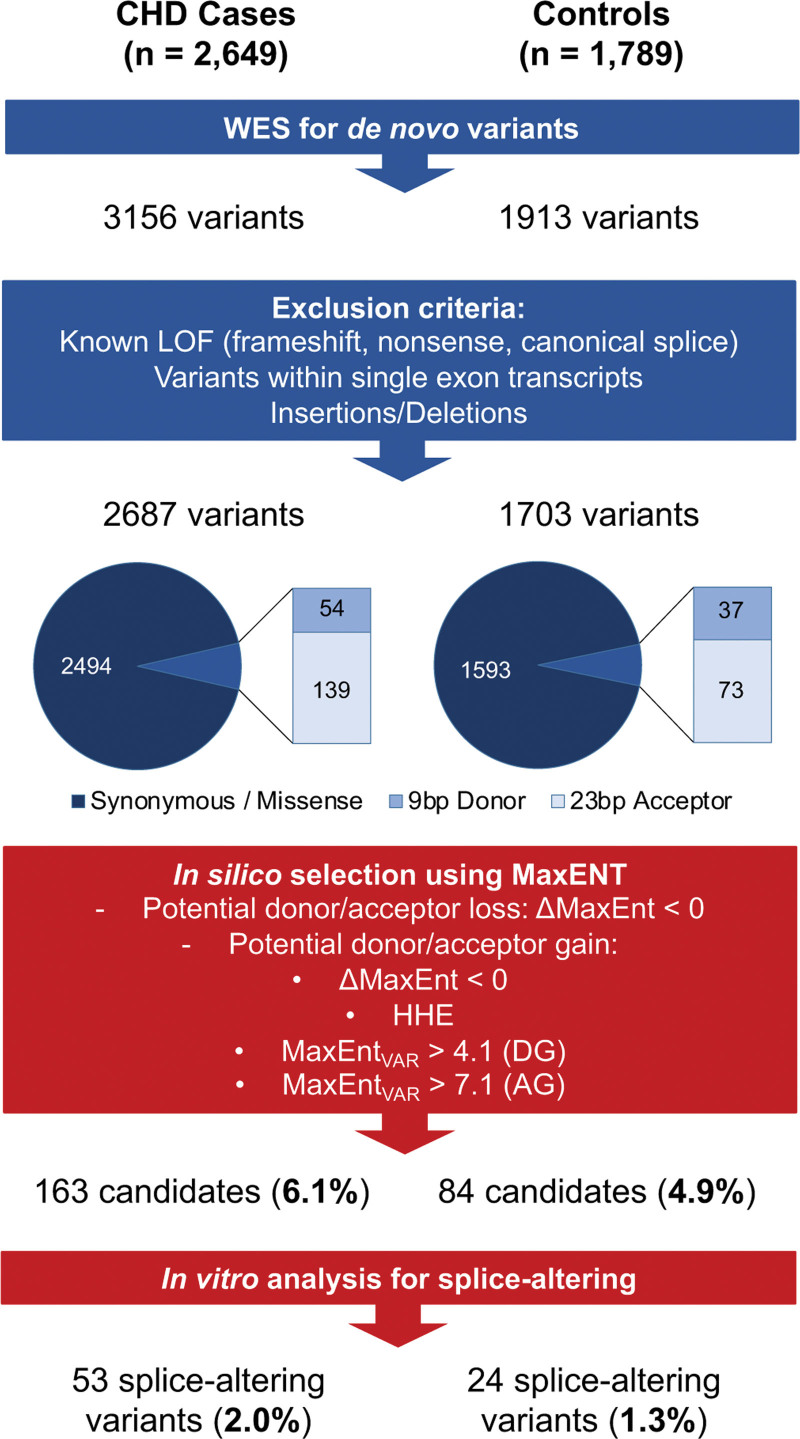
**Schematic of the analyses of putative de novo RNA splice-altering variants found in congenital heart disease (CHD) trios and controls.** From whole exome sequencing (WES) of CHD trios (n=2649) and controls (n=1789), de novo single-nucleotide variants were filtered to exclude coding and canonical splice sequences predicted to cause loss of function (LoF). Computated MaxENT scores (Methods) identified variants (163 in CHD cases and 84 in controls) that were further prioritized for functional analyses. Splice assays in minigene constructs identified 53 splice-altering variants in CHD cases and 24 splice-altering variants in controls. HHE indicates high heart expression denoting the top quartile of transcripts expressed in the e14.5 mouse heart.

Using computational predictions based on the MaxENT score (Supplemental Methods), we prioritized 163 of 193 (84.5%) variants in the CHD cohort and 84 of 110 (76.4%) variants in controls for confirmation in in vitro splice assays. Minigenes assays showed that 53 of 163 (32.5% and 2.0% of computationally identified variants) variants in CHD probands and 24 of 84 (28.6% and 1.3% of identified variants) variants in controls caused significant splicing differences in comparison to reference sequences (Figure 1; Table S1). As prior WES analyses^6^ identified 366 de novo LoF variants in the CHD cohort, the addition of 53 splice-altering variants represented a 15.3% increase. In the Simons Foundation Autism Research Initiative controls, we identified 24 de novo splice variants in addition to 146 previously identified de novo LoF variants, representing a 16.4% increase (Table S2). Identification of new splice-altering variants equally increased the proportion of LoF variants in each cohort.

We considered whether these splice-altering variants altered 1 of 253 genes that have established or plausible roles in CHD based on analyses of patients and model organisms (Table S3).^6,7^ Two of 4 splice-altering variants identified in the CHD cohort altered *KMT2D*, causing Kabuki syndrome and 1 each in *RSP24*, causing Diamond-Blackfan anemia and in *RBFOX2*, a gene recurrently mutated in children with hypoplastic left heart syndrome (Table 1). Each of these genes are highly expressed in the heart and previously described as dominant-acting CHD disease genes.^13,14^ Among the 24 splice-altering variants found in the control cohort, 1 variant is in a recessive CHD gene, *CNTRL*, encoding a centriole-appendage protein centriolin. *CNTRL* is not highly expressed in the heart (Table 1). There was no increased burden of RNA splice-altering DNVs in CHD cases versus controls.

**Table 1.**
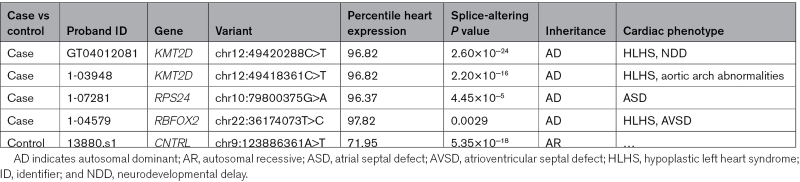
Splice-Altering De Novo Variants in Congenital Heart Disease Genes in Cases and Controls

### Rare Splice-Altering Variants in CHD Genes Found in Probands and Controls

**Figure 2.**
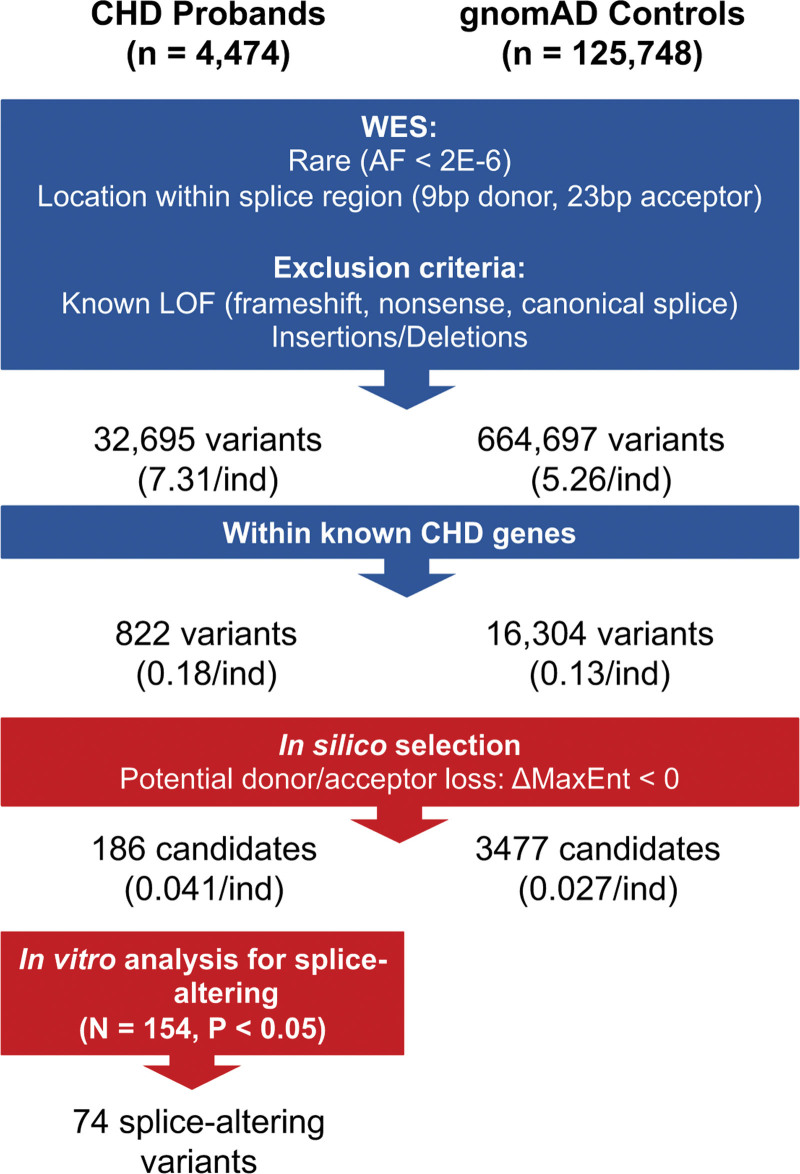
**Schematic of the analyses of putative rare RNA splice-altering variant candidates in congenital heart disease (CHD) probands and gnomAD controls.** We restricted functional analyses to rare (allele frequency [AF], <2×10^−^^6^ in gnomAD) single-nucleotide variants residing within 9 bp of donor and 23 bp acceptor regions of CHD genes in 4474 CHD and 125 748 gnomAD controls. Based on computational prioritization of variants likely to cause the loss of donor or acceptor splice (calculated ΔMaxEnt score, <0), we studied 186 (22.6%) using an in vitro splicing assay and identified 74 as splice altering. Parallel processing of variants in gnomAD controls identified 664 697 variants within splice sequences, of which 16 304 were encoded in CHD genes. Computational prioritization using MaxENT scores identified 3477 candidate variants (2301 within 9 bp of 5′ donor splice sequences and 1176 within the 23 bp of 3′ acceptor sequences). gnomAD candidate variants were not assessed in the minigene assay.

We considered whether rare variants (allele frequency, <2×10^−^^6^) that may be inherited might also alter splicing, by analyzing an expanded CHD cohort (4474 probands, inclusive of 2649 probands from trios) and 125 748 gnomAD controls. We filtered variants as described above and further considered only rare variants within 5′ss and 3′ss that are predicted to cause donor or acceptor loss, as our prior splicing assays could confirm more splice site losses than gains.^10,11^ These criteria yielded 32 695 variants in CHD and 664 697 variants in the gnomAD controls (Figure 2). We further restricted analyses to variants within the 253 CHD genes.^7^ This resulted in a final list of 822 variants in CHD probands (0.18 per person) and 16 304 variants in controls (0.13 per person).

Computational selection (Supplemental Methods) yielded 186 candidate variants in CHD probands and 3477 candidate variants in controls. Functional assays were performed on the candidate variants from CHD probands. We identified 74 variants that significantly altered splicing when compared with reference sequence (Figure 2; Table S4).

### Computational Selection of Likely Splice-Altering Variants Using SpliceAI

We assessed the performance of SpliceAI,^8^ a computational predictive algorithm that classifies variants as splice altering or benign, by comparing predictions with results from splicing assays using minigene constructs. We assessed 334 variants inclusive of 200 DNVs and 134 rare variants using SpliceAI with parameters to yield high recall (cutoff, ≥0.2) and high precision (cutoff, ≥0.8) for predicting acceptor and donor loss and acceptor and donor gain (Table 2) and required a significant difference (*P*<0.05) in splice assays containing the variant and reference sequence (Methods). At the lower cutoff, SpliceAI had a positive predictive value (PPV) and negative predictive value (NPV) of 77.3% and 74.0%, respectively, with a sensitivity of 0.66 and specificity of 0.84. The more stringent SpliceAI parameter increased the PPV to 93.8% and decreased in NPV to 59.1%, with a decrease in sensitivity to 0.20 and corresponding increase in specificity to 0.99. When limiting the analysis to acceptor and donor loss, SpliceAI (cutoff, ≥0.2) yielded PPV of 80.9% and NPV of 68.9% with sensitivity of 0.65 and specificity of 0.84. SpliceAI (cutoff, ≥0.8) yielded PPV of 100% and NPV of 54.0%, with sensitivity of 0.20 and specificity of 1.

**Table 2.**
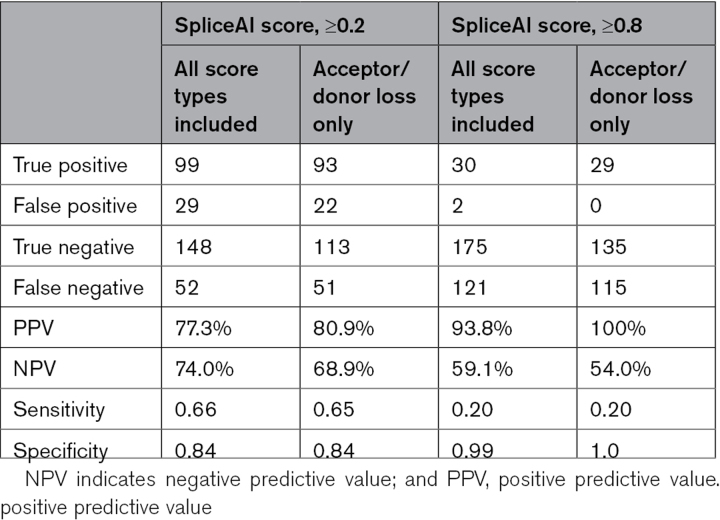
Performance of SpliceAI Among All Variants Assessed by Minigenes Assay

### Increased Burden of Rare RNA Splice-Altering Variants in CHD Probands

We then considered the burden of predicted rare splice-altering variants within 253 CHD genes in probands and controls using both computational predictive algorithms, MaxENT scores (Methods), and SpliceAI^8^ (cutoff, ≥0.8). MaxENT scores identified 0.042 rare splice-altering variants per CHD proband compared with 0.028 rare splice-altering variants per gnomAD individual, a 50.4% increase (*P*=6.94×10^−^^8^). SpliceAI similarly predicted a significantly increased burden (*P*=1.28×10^−^^4^) of rare splice-altering variants in the CHD cohort (n=38; 0.0095 variants per proband) compared with gnomAD (n=552; 0.0044 variants per individual).

### Noncanonical Splice Variants Inform Genetic Pathogeneses in CHD Probands

Our analyses yielded 127 splice-altering variants (53 DNVs, 74 rare) in CHD probands. Among these, 4 DNVs and 74 rare variants reside in established or candidate CHD genes, including 31 variants that are within previously identified dominant CHD genes (Table S5).

Each of these 78 newly identified, splice-altering variants in CHD genes occurred in a unique proband. Among these 78 probands, 9 probands had a previously identified de novo or rare LoF variant^6,7^ (Table 3). Two of these 9 LoF variants were in dominant CHD genes and, therefore, likely pathogenic (Table 3, proband GT04008173 and proband 1-01026). As splice-altering variants are likely to cause LoF, these provide an alternative or complementary genetic pathogenesis for CHD in 69 probands (≈1.5% of the cohort). The clinical phenotypes of these probands were consistent with clinical manifestations associated with LOF variants in these CHD genes (Table S5).

**Table 3.**
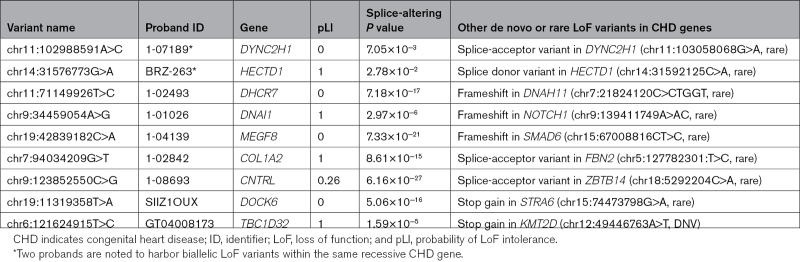
Probands With Both a Splice-Altering and LoF Coding Variant in CHD Genes

**Figure 3.**
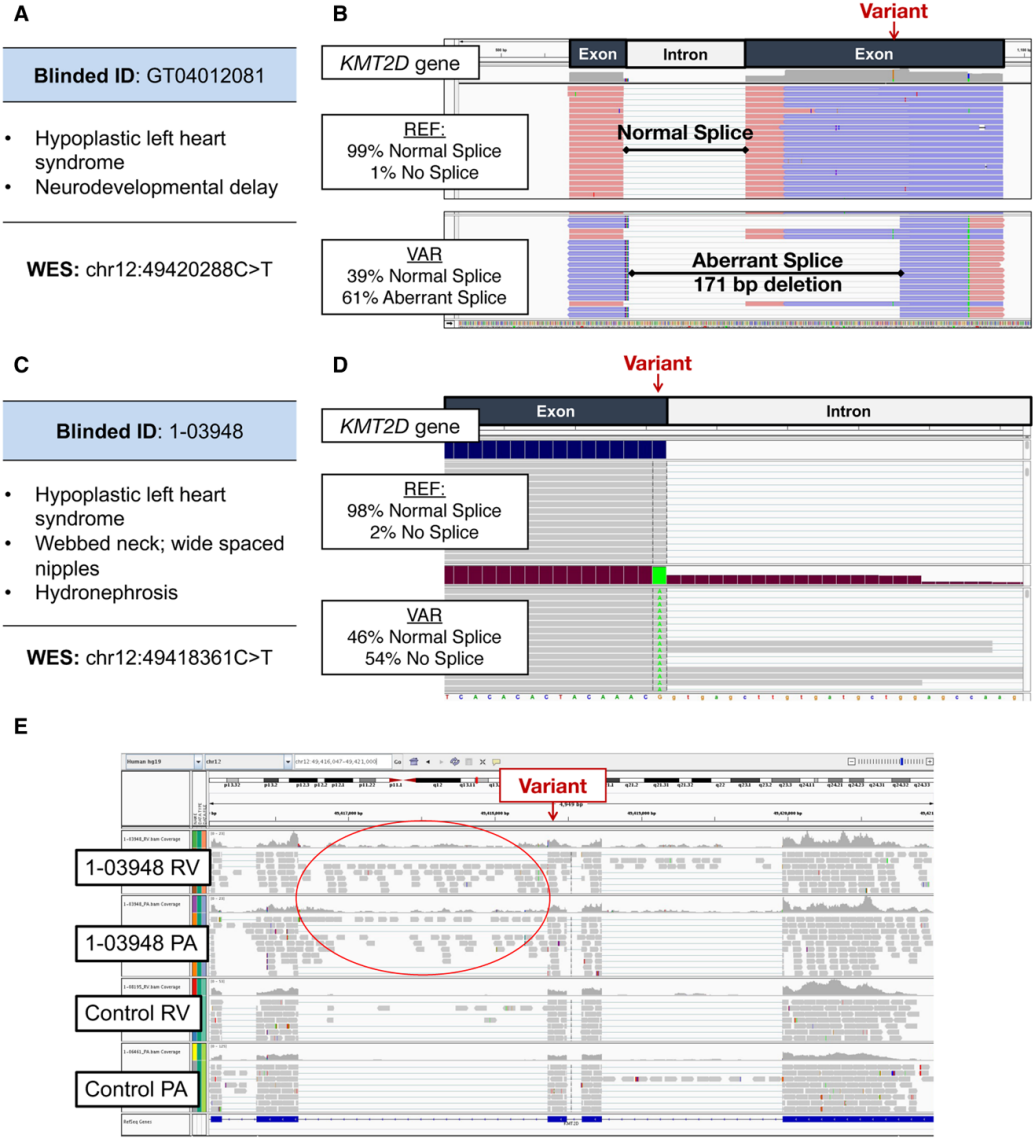
**RNA splice-altering variants in *KMTD2* identified by splice assays and confirmed by cardiac tissue confirmation. A**, Clinical and genetic profiles of congenital heart disease (CHD) proband GT04012081. The identified exon 48 variant in *KMT2D* encodes a missense residue with unknown impact on protein function or clinical significance. **B**, Computational analyses (MaxENT scores) and in vitro RNA splicing assay demonstrated that the variant produces an inappropriate 3′ splice site acceptor, resulting in a 171-base pair deletion in the minigene and is predicted to encode a truncated protein due to a frameshift. **C**, Clinical and genetic profiles of CHD proband 1-03948 identified a variant in *KMT2D* in exon 50 in the 5′ss, adjacent to the canonical splice site. **D**, Computational analyses and splicing assay indicated loss of normal splicing with read through of intron sequences. **E**, RNA sequencing of right ventricular (RV) and pulmonary artery (PA) samples from 1-03948, compared with RV and PA tissues from CHD proband with normal *KMT2D* sequence (control) confirms aberrant splicing, in vivo with retention of intron sequences.

In addition, a subset of splice-altering variants identified in CHD genes of these probands occurred in CHD genes associated with established clinical syndromes. Four novel splice-altering variants were identified in *KMT2D* (2 rare, 2 DNV). Haploinsufficiency of *KMT2D* causes Kabuki syndrome, which is characterized by craniofacial abnormalities, neurodevelopmental delay, and CHD.^15^ Proband GT04012081 with hypoplastic left heart syndrome and neurodevelopmental delay was clinically diagnosed with Kabuki syndrome but without a genetic pathogenesis. The proband has a potentially damaging de novo *KMT2D* missense mutation (chr12:49420288C>T) that substitutes a glycine for arginine residue (Figure 3A). However, the computational prediction and splice assay confirmed that this variant results in a splice site acceptor gain, with significantly stronger activity than the reference sequence (*P*=2.6×10^−24^). Aberrant splicing of the minigene construct demonstrates that this variant produces a novel splice-acceptor site resulting in 171-base-pair deletion of minigene sequence (Figure 3B). The processed RNA is predicted to encode truncated *KMT2D*.

Proband 1-03948, also with hypoplastic left heart syndrome and syndromic features of Kabuki syndrome, carried a DNV in exon 50 of *KMT2D* (chr12:49418361C>T; Figure 3C), located at the −1 position adjacent to the canonical GT donor site. This variant caused abnormal splicing (*P*=2.2×10^−^^16^ versus reference) with retention of intronic sequences in the minigene construct (Figure 3D). Using surgically discarded right ventricle (RV) and pulmonary artery (PA) tissues from this proband, we isolated and sequenced *KMT2D* transcripts. Like the mis-spliced minigene construct, these showed high levels (RV, 25%; PA, 30%) of retained intron adjacent to the variant (Figures 3D and 3E). Using discarded RV (n=109) and PA (n=39) tissues from other CHD probands, we calculated the inclusion and exclusion of intron sequences. The median percentage of intron spliced in for the RV and PA was 5.3% and 9.3%, respectively. These control data, fitted to a linear (r_RV_^2^, 0.4; r_PA_^2^, 0.1), indicate that proband 1-03948 had a significant deviation from the expected ratio (calculated as the orthogonal projection onto the linear model) in both RV (*Z* score, 4.5; *P*=3.7×10^−6^) and PA (*Z* score, 1.9; *P*=0.03). Collectively, we suggest that these data provide strong evidence for pathogenicity of this *KMT2D* noncanonical splice variant.

Our studies combined with prior analyses also uncovered 2 CHD probands who carried biallelic LoF variants in recessive CHD genes (Table 3). Proband 1-07189 carries both a rare, canonical splice-acceptor site variant (chr11:103058068G>A) in *DYNC2H1* and a second variant (chr11:102988591A>C) that significantly altered splicing in comparison to reference sequences (*P*=7.05×10^−3^) that is classified by ClinVar as a variant of uncertain significance. *DYNC2H1* encodes a recessive ciliary gene that causes short rib thoracic dysplasia, with and centriole-appendage protein without polydactyly and additional malformations including CHD.^16^ Proband 1-07189 has an atrioventricular canal defect without extracardiac manifestations.

Proband BRZ-263 has 2 variants in *HECTD1*, a rare, canonical splice donor variant (chr14:31592125C>A) and another (chr14:31576773G>A) that was a splice-altering variant in our assays (*P*=0.0278 versus reference sequence). As *HECTD1* encodes a ubiquitin ligase that participates in aortic arch development,^17^ these biallelic *HECTD1* variants are plausible causes for tetralogy of Fallot and an aortic arch defect in proband BRZ-263.

## Discussion

Here, we considered the potential for variants in noncoding sequences in proximity to canonical splice signals to disrupt normal RNA processing and contribute to unexplained CHD. We demonstrate that about 1.5% of patients with CHD have a rare noncanonical splice-altering variant in a CHD gene that is likely responsible for their disease. Both familial segregation of CHD with an inherited damaging coding variants and the co-occurrence of sporadic CHD with damaging DNVs provides strong support for causality. The combination of computational and in vitro splicing assays defined adverse functional consequences of noncoding DNVs and rare variants in WES data sets. Among a CHD cohort comprised of 4474 probands, we identified 53 de novo and 74 rare variants that perturbed splicing of a minigene construct, indicating that these are damaging and likely to cause LoF. Of these 123 variants, 78 variants altered genes with previously defined, damaging coding variants that cause CHD. Among the 78 CHD probands with splice-altering variants, only 2 had prior genetic diagnoses. The additional analyses of noncanonical splice sequences provided a likely genetic explanation for CHD in ≈1.5% of probands.

Splice-altering variants altered dominant-acting CHD genes including *KMT2D*, *RBFOX2*, and *RPS24*. The clinical phenotypes of probands with these variants are similar to those previously reported with LOF mutations in these genes (Table S5). Additionally, our data uncovered biallelic variants in recessive genes such as *DYNC2H1*, which expands our understanding of the genetic architecture of CHD. While we identified splice-altering variants in other recessive genes, definitive evidence for their pathogenicity requires the identification of a second damaging variant. Genome sequencing and analyses of other noncoding variants may provide these data.

Our splice assays also provided an assessment of SpliceAI, a new machine learning–based tool for predicting the impact of variants on splicing. SpliceAI had a high PPV (≈93%) when using a stringent cutoff, a finding that is consistent with our previous assessment of this tool for analyzing cardiomyopathy genes.^12^ The PPV increased to 100% when applied only to variants that are predicted to cause loss of the splice site. However, as SpliceAI had an NPV of 60% to 70%, cautious use of computational tools alone is necessary as these can miss potentially damaging variants that are uncovered by functional interrogation using an in vitro system or ex vivo tissue analyses.

We recognize several limitations in our study. Thirty-one of 433 total candidate variants, often occurring in repetitive regions, could not be tested with minigenes because the required oligonucleotides could not be synthesized. In addition, ≈17% of minigenes carrying variants yielded indeterminate results (Tables S1 and S4). Although our RNA sequencing of heart tissue from one CHD proband confirmed the effects detected by the splice assay, we recognize that variants may impact splicing or other aspects of RNA processing that are not captured by the in vitro assay. We did not assess the potential for a novel splicing event that might increase splicing in CHD genes.^18,19^ We also did not assess the possible contribution of these rare splice-altering variants to a multigenic pathogenesis of CHD. Finally, we interrogated sequences flanking canonical splice sequences, where sequences beyond these regions, including splice enhancers and silencers, and sequences involved in spliceosome and RNA binding protein interactions were not assessed.

In summary, assessing the ability of rare and DNVs found in CHD probands to alter RNA splicing yielded likely pathogenic variants and contributed molecular diagnoses to probands with unexplained CHD. These results along with pathogenic variants in canonical splice signals indicate that disruption in normal RNA splicing is an important contributory mechanism for the pathogenesis of CHD. Future improvements in genome-based precision medicine for genetic diagnosis of CHD should include evaluation of noncanonical splice sequences in known CHD genes. Extending these analyses is expected to uncover additional pathogeneses for unexplained CHD and other disorders.

## Article Information

### Acknowledgments

The authors would like to express their gratitude to all the patients and their families.

### Sources of Funding

This work was supported by the National Heart, Lung, and Blood Institute grants for the Pediatric Congenital Genomics Consortium (U01-HL098188, U01-HL131003, UM1-HL098147, U01-HL098153, U01-HL098163, UM1-HL098123, UM1-HL098162, UM1-HL128761, and UM1-HL128711). Dr Young Jang was supported by the Howard Hughes Medical Institute Med Fellows Program. Dr Patel was supported by a fellowship from the Sarnoff Cardiovascular Research Foundation. Drs Pereira, Morton, and Seidman were supported by NHLBI R03-HL150412-01A1.

### Disclosures

None.

### Supplemental Material

Supplemental Methods

Tables S1–S5

Figure S1

References 20–22

## Supplementary Material

## References

[1] Hoffman JIE Kaplan S. The incidence of congenital heart disease. J Am Coll Cardiol. 2002;39:1890–1900. doi: 10.1016/s0735-1097(02)01886-7 12084585 10.1016/s0735-1097(02)01886-7

[2] Calzolari E Garani G Cocchi G Magnani C Rivieri F Neville A Astolfi G Baroncini A Garavelli L Gualandi F. Congenital heart defects: 15 years of experience of the Emilia-Romagna Registry (Italy). Eur J Epidemiol. 2002;18:773–780. doi: 10.1023/a:1025312603880 10.1023/a:1025312603880 12974553

[3] Fahed AC Gelb BD Seidman JG Seidman CE. Genetics of congenital heart disease: the glass half empty. Circ Res. 2013;112:707–720. doi: 10.1161/CIRCRESAHA.112.300853 23410880 10.1161/CIRCRESAHA.112.300853 PMC3827691

[4] Zaidi S Brueckner M. Genetics and genomics of congenital heart disease. Circ Res. 2017;120:923–940. doi: 10.1161/CIRCRESAHA.116.309140 28302740 10.1161/CIRCRESAHA.116.309140 PMC5557504

[5] Morton SU Quiat D Seidman JG Seidman CE. Genomic frontiers in congenital heart disease. Nat Rev Cardiol. 2022;19:26–42. doi: 10.1038/s41569-021-00587-4 34272501 10.1038/s41569-021-00587-4 PMC9236191

[6] Jin SC Homsy J Zaidi S Lu Q Morton S Depalma SR Zeng X Qi H Chang W Sierant MC. Contribution of rare inherited and de novo variants in 2,871 congenital heart disease probands. Nat Genet. 2017;49:1593–1601. doi: 10.1038/ng.3970 28991257 10.1038/ng.3970 PMC5675000

[7] Homsy J Zaidi S Shen Y Ware JS Samocha KE Karczewski KJ DePalma SR McKean D Wakimoto H Gorham J. De novo mutations in congenital heart disease with neurodevelopmental and other congenital anomalies. Science. 2015;350:1262–1266. doi: 10.1126/science.aac9396 26785492 10.1126/science.aac9396 PMC4890146

[8] Jaganathan K Kyriazopoulou Panagiotopoulou S McRae JF Darbandi SF Knowles D Li YI Kosmicki JA Arbelaez J Cui W Schwartz GB. Predicting splicing from primary sequence with deep learning. Cell. 2019;176:535–548.e24. doi: 10.1016/j.cell.2018.12.015 30661751 10.1016/j.cell.2018.12.015

[9] Caminsky N Mucaki EJ Rogan PK. Interpretation of mRNA splicing mutations in genetic disease: review of the literature and guidelines for information-theoretical analysis. F1000Res. 2014;3:282. doi: 10.12688/f1000research.5654.1 25717368 10.12688/f1000research.5654.1 PMC4329672

[10] Ito K Patel PN Gorham JM McDonough B DePalma SR Adler EE Lam L MacRae CA Mohiuddin SM Fatkin D. Identification of pathogenic gene mutations in LMNA and MYBPC3 that alter RNA splicing. Proc Natl Acad Sci USA. 2017;114:7689–7694. doi: 10.1073/pnas.1707741114 28679633 10.1073/pnas.1707741114 PMC5528995

[11] Patel PN Gorham JM Ito K Seidman CE. In vivo and In vitro methods to identify DNA sequence variants that alter RNA splicing. Curr Protoc Hum Genet. 2018;97:e60. doi: 10.1002/cphg.60 30038698 10.1002/cphg.60 PMC6054316

[12] Patel PN Ito K Willcox JAL Haghighi A Jang MY Gorham JM DePalma SR Lam L McDonough B Johnson R. Contribution of noncanonical splice variants to TTN truncating variant cardiomyopathy. Circ Genom Precis Med. 2021;14:e003389. doi: 10.1161/CIRCGEN.121.003389 34461741 10.1161/CIRCGEN.121.003389 PMC8788938

[13] Adam MP Hudgins L. Kabuki syndrome: a review. Clin Genet. 2005;67:209–219. doi: 10.1111/j.1399-0004.2004.00348.x 15691356 10.1111/j.1399-0004.2004.00348.x

[14] Boria I Garelli E Gazda HT Aspesi A Quarello P Pavesi E Ferrante D Meerpohl JJ Kartal M Costa LD. The ribosomal basis of diamond-blackfan anemia: mutation and database update. Hum Mutat. 2010;31:1269. 20960466 10.1002/humu.21383 PMC4485435

[15] Miyake N Mizuno S Okamoto N Ohashi H Shiina M Ogata K Tsurusaki Y Nakashima M Saitsu H Niikawa N. KDM6A point mutations cause Kabuki syndrome. Hum Mutat. 2013;34:108–110. doi: 10.1002/humu.22229 23076834 10.1002/humu.22229

[16] Cheng C Li X Zhao S Feng Q Ren X Chen X. Compound heterozygous variants in DYNC2H1 in a foetus with type III short rib-polydactyly syndrome and situs inversus totalis. BMC Genom. 2022;15:55. 10.1186/s12920-022-01205-z PMC8917749 35277174

[17] Sugrue KF Sarkar AA Leatherbury L Zohn IE. The ubiquitin ligase HECTD1 promotes retinoic acid signaling required for development of the aortic arch. Dis Model Mech. 2019;12(1):1–12. 10.1242/dmm.036491 PMC6361158 30578278

[18] Baralle FE Giudice J. Alternative splicing as a regulator of development and tissue identity. Nat Rev Mol Cell Biol. 2017;18:437–451. doi: 10.1038/nrm.2017.27 28488700 10.1038/nrm.2017.27 PMC6839889

[19] Scotti MM Swanson MS. RNA mis-splicing in disease. Nat Rev Genet. 2016;17:19–32. doi: 10.1038/nrg.2015.3 26593421 10.1038/nrg.2015.3 PMC5993438

[20] Iossifov I O’Roak BJ Sanders SJ Ronemus M Krumm N Levy D Stessman HA Witherspoon KT Vives L Patterson KE. The contribution of de novo coding mutations to autism spectrum disorder. Nature. 2014;515:216–221. doi: 10.1038/nature13908 25363768 10.1038/nature13908 PMC4313871

[21] Karczewski KJ Francioli LC Tiao G Cummings BB Alföldi J Wang Q Collins RL Laricchia KM Ganna A Birnbaum DP; Genome Aggregation Database Consortium. The mutational constraint spectrum quantified from variation in 141,456 humans. Nature. 2020;581:434–443. doi: 10.1038/s41586-020-2308-7 32461654 10.1038/s41586-020-2308-7 PMC7334197

[22] Zaidi S Choi M Wakimoto H Ma L Jiang J Overton JD Romano-Adesman A Bjornson RD Breitbart RE Brown KK. De novo mutations in histone-modifying genes in congenital heart disease. Nature. 2013;498:220–223. doi: 10.1038/nature12141 23665959 10.1038/nature12141 PMC3706629

