# Supplementary material for: Contribution of Previously Unrecognized RNA Splice-Altering Variants to Congenital Heart Disease
Source: Circ Genom Precis Med. 2023 May 11;16(3):224–31. doi: 10.1161/CIRCGEN.122.003924 (PMC10404383; doi:10.1161/CIRCGEN.122.003924)
Supplement: Supplementary file 1 [file hcg-16-224-s001.pdf]

SUPPLEMENTAL MATERIAL

Supplemental Methods:

We studied *de novo* variants (DNVs) within splice regions, including the 9-base pairs (bp) located 5' splice donor site (5'ss) and 23-bp located 3' splice acceptor site (3'ss) that were identified from WES analyses of two cohorts. The PCGC DNVs were identified in 2,649 CHD trios, comprised of parents and affected offspring, who were recruited and sequenced by the Pediatric Congenital Genomics Consortium (PCGC) of the National Heart, Lung, and Blood Institute (NHLBI)[6]. The SFARI DNVs were identified in 1,789 trios, comprised of unaffected parents and an unaffected sibling of a child with autism, were recruited and sequenced by the Simons Foundation Autism Research Initiative (SFARI) Simplex Collection[21].

We also analyzed rare variants (allele frequency (AF) ≤ 2E-6 in the Genome Aggregation Database (gnomAD); https://gnomad.broadinstitute.org/) within 5'ss and 3'ss from WES data obtained on 4,474 CHD probands, inclusive of 2,649 trio probands and isolated probands, that were recruited and sequenced by the PCGC and in 125,748 individuals reported in the gnomAD database[22].

Variant selection and prioritization using MaxENT

After excluding DNVs predicted to cause LoF, including frameshifts, nonsense, insertions/deletions, and canonical splice site alterations and all variants within single-exon transcripts, we categorized variants by location within the 5'ss or 3'ss regions, or as synonymous and missense coding variants. We prioritized these variants for study using the previously described computational algorithm, MaxENT with Regress_Score.v.095.R, applying filters that are most likely to identify splice-altering variants[10,11]. For potential donor or acceptor loss candidates, the calculated ΔMaxEnt scores were < 0. For variants outside the 5'ss and 3'ss,

inclusive of coding variants, potential donor gain (DG) had a calculated ΔMaxEnt > 0 and $MaxEnt_{VAR}$ > 4.1 and for potential acceptor gain (AG), the calculated $MaxEnt_{VAR}$ > 7.1. We only assessed DG and AG variants within genes with high heart expression (HHE), defined as the top quartile of expression from RNA sequencing of mouse heart at embryonic day 14.5[23].

For rare variants analyses, we considered only single-nucleotide variants (SNVs) within the 5'ss and 3'ss regions. As 32,695 PCGC and 664,697 gnomAD rare variants were identified, we studied only variants within a set of 253 CHD genes (Supplemental Table III)[7] and that are predicted to cause donor loss or acceptor loss (ΔMaxENT < 0 with Regress_Score.v.095.R), as our earlier functional assays[11] more consistently confirmed these in comparison to DG and AGs.

Minigene design, synthesis, and construction

Using the Construct_design.R tool[11] we designed paired minigene constructs (≤ 500 bp) with the variant (ALT) or reference (REF) splice sequence, an exon with the donor site, a truncated intron, an exon containing the acceptor site, and a 2-bp barcode sequence to allow for multiplexing. After synthesis, (Integrated DNA Technologies or TWIST Biosciences), minigenes were PCR-amplified and purified, and both a CMV promoter and poly-A tail were added[10], resulting in constructs ~1,200 bp in size (Supplemental Figure I).

In vitro assay using HEK cells and RNA sequencing

Pooled minigene constructs (n= 20; 100 ng) were transfected into HEK293T cells using Lipofectamine 2000 (Thermo Fisher), concurrently with the pMaxGFP plasmid to confirm successful transfection. After 24 hours, RNA was collected in TRIzol (Thermo Fisher), isolated using phenol-chloroform extraction, and quality assessed by calculating the RNA integration

number equivalent (RIN) using Agiliant Tapestation 3000. Samples with RIN > 9.0 were used to construct cDNA libraries using SuperScript III (Thermo Fisher) and prepared for sequencing using the Illumina MiSeq platform.

Sequencing analysis and burden analysis

Sequences were processed to trim adapters and pairs were matched using FLASH (https://ccb.jhu.edu/software/FLASH/) using previously published scripts (located at https://GitHub.com/Splicing Variant/SplicingVariants_Beta). We assessed splicing only when sequence data contained greater than 100 reads for both REF and ALT constructs, and REF constructs showed greater than 10% normal splicing. Variants within constructs that did not meet these criteria were considered "indeterminate" while others were classified as normal splice, no splice, or aberrant splice. Each splice outcome was normalized to 100 and ratios were calculated for aberrant splicing vs. normal splicing. We calculated P-values using Fisher's exact test to compare ratios of REF and ALT constructs and considered $P < 0.05$ as significant.

Burden analyses for splice variants in CHD cohorts compared to control cohorts were performed by calculating the number of variants per individual in each cohort and comparing these ratios using right-tailed binomial test.

RNAseq of cardiac tissue

RNA was obtained from discarded pulmonary artery and right ventricular tissues obtained during surgery from CHD probands. CHD probands were recruited from two centers into the Congenital Heart Disease Genetic Network Study of the Pediatric Cardiac Genomics Consortium (CHD Genes: NCT01196182). The protocol was approved by the Institutional

Review Boards of Boston Children's Hospital, Brigham and Women's Hospital, Great Ormond St Hospital, Children's Hospital of Los Angeles, and Yale School of Medicine. Written informed consent was obtained from each participating subject or parent/guardian. RNA was extracted using the phenol-chloroform method and solubilized in nuclease-free water. RNA (RNA integrity index (RIN) >8.0) was quantified (Agilent TapeStation 2200), and RT-PCR was processed (using a Nextera kit) for sequencing on an Illumina HiSeq or NextSeq. 20-30 million reads were obtained per RNAseq library.

Computational Prioritization of Variants using SpliceAI

We also used the SpliceAI algorithm, applying scores reported to yield high recall (0.2) and high precision (0.8) thresholds[8] to assess rare variants in CHD probands and gnomAD controls. We assessed the positive predictive values (PPV) and negative predictive values (NPV) of SpliceAI in CHD samples studied by splice assays and classified variants as true positives and true negatives when SpliceAI predictions and minigenes assays agreed. False positive denotes variants predicted by SpliceAI to cause abnormal splicing that were not confirmed by minigenes assays. Conversely, false negatives variants denote those predicted to have no effect by SpliceAI but that altered splicing in minigenes assay.

Supplemental Table I: Extended results of all tested variants/gene blocks for *de novo* variants in cases and controls (provided in separate Excel spreadsheet)

Supplemental Table II: Increased yield of whole exome sequencing using splicing assay

	**Cases (n = 2,649)**	**Controls (n = 1,789)**
**LoF – Whole Exome Sequencing**	366	146
**Splice Altering Variants**	53	24
**% Increase**	**15.3%**	**16.4%**

Supplemental Table III: List of human/mouse CHD genes (provided in separate Excel spreadsheet)

Supplemental Table IV: Extended results of all tested variants/gene blocks for rare variants in cases and controls (provided in separate Excel spreadsheet)

Supplemental Table V: Clinical phenotypes of probands with newly identified LoF RNA splice-altering variants (provided in separate Excel spreadsheet)

Supplemental Figure I: Overview of Minigene splice assay

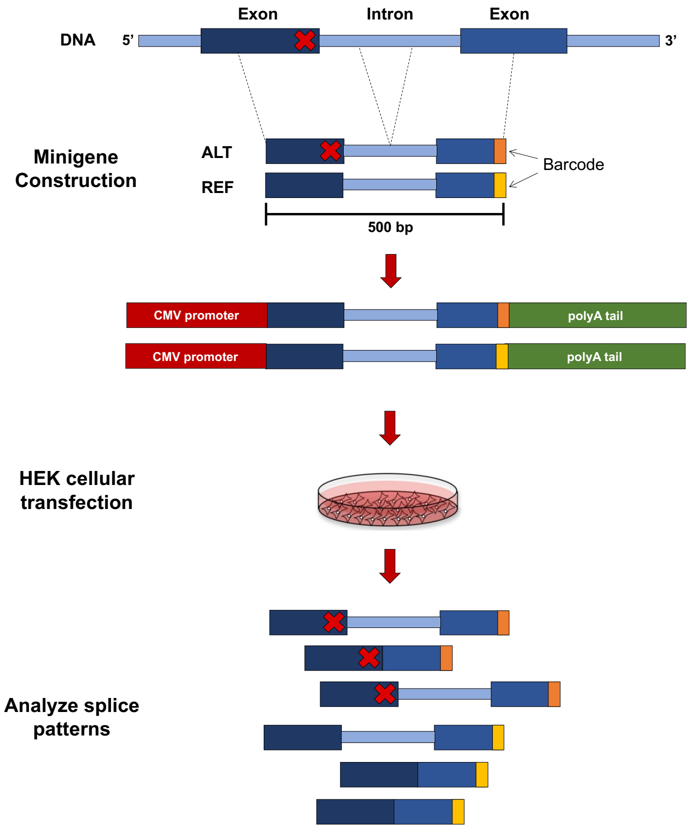

This two-part assay is composed of a computational component that assess the ability for each variant to alter RNA splicing, and an *in vitro* component using synthesized Minigene constructs. Computationally selected variants are synthesized into a Minigene construct, which consists of the abbreviated exon-intron-exon junction, containing both the 5' 9-bp splice donor site as well as the 3' 23-bp splice acceptor site. Both a reference Minigene (REF) and Minigene containing the variant (ALT) are constructed, and a distinct 2-bp barcode is attached to each. These Minigenes are then pooled and transfected into HEK293T cells. Subsequently, the RNA is extracted and sequenced with MiSeq. Sequence analysis allows assessment of the selected variants' impact on the resultant transcript.
